# Impact of HIV treat-all and complementary policies on ART linkage in 13 PEPFAR-supported African countries

**DOI:** 10.1186/s12913-023-09702-2

**Published:** 2023-10-25

**Authors:** Anna Russell, Andre R. Verani, Sherri Pals, Valamar M. Reagon, Lorraine N. Alexander, Eboni T. Galloway, Mayer Magdalene Mange, Pearl Kalimugogo, Ponesai Nyika, Yasmine Moussa Fadil, Appolonia Aoko, Fred Mugyenyi Asiimwe, Akudo Ikpeazu, Dumbani Kayira, Mpho Letebele, Alice Maida, Daniel Magesa, Gram Mutandi, Annie C. Mwila, Dennis Onotu, Kingsly Tse Nkwoh, Evelyn Wangari

**Affiliations:** 1 Independent Researcher (formerly Centers for Disease Control & Prevention), 1600 Clifton Rd, GA 30333 Atlanta, USA; 2 Division of Global HIV & TB, Centers for Disease Control & Prevention (CDC), Atlanta, USA; 3 Commissioned Corps, United States Public Health Service, Atlanta, USA; 4 Division of Global HIV and TB, Center for Global Health, CDC, Yaoundé, Cameroon; 5 Division of Global HIV and TB, Center for Global Health, CDC, Windhoek, Namibia; 6 Division of Global HIV and TB, Center for Global Health, CDC, Harare, Zimbabwe; 7 Division of Global HIV and TB, Center for Global Health, CDC, Nairobi, Kenya; 8 Division of Global HIV and TB, Center for Global Health, CDC, Maseru, Lesotho; 9 Sexually Transmitted Infections Control and Hepatitis Program (NASCP), National AIDS, Federal Ministry of Health, Abuja, Nigeria; 10 Division of Global HIV and TB, Center for Global Health, CDC, Lilongwe, Malawi; 11 Division of Global HIV and TB, Center for Global Health, CDC, Gaborone, Botswana; 12 Division of Global HIV and TB, Center for Global Health, CDC, Dar es Salaam, Tanzania; 13 Division of Global HIV and TB, Center for Global Health, CDC, Lusaka, Zambia; 14 Division of Global HIV and TB, Center for Global Health, CDC, 1600 Clifton Rd, GA 30333 Atlanta, Nigeria

**Keywords:** HIV/AIDS, Linkage, Retention, Treat-all, Test and start

## Abstract

**Background:**

In 2015, the World Health Organization recommended that all people living with HIV begin antiretroviral treatment (ART) regardless of immune status, a policy known as ‘Treat-All to end AIDS’, commonly referred to as Treat-All. Almost all low- and middle-income countries adopted this policy by 2019. This study describes how linkage to treatment of newly diagnosed persons changed between 2015 and 2018 and how complementary policies may have similarly increased linkage for 13 African countries. These countries adopted and implemented Treat-All policies between 2015 and 2018 and were supported by the U.S. Government’s President’s Emergency Plan for AIDS Relief (PEPFAR). The focuses of this research were to understand 1) linkage rates to ART initiation before and after the adoption of Treat-All in each country; 2) how Treat-All implementation differed across these countries; and 3) whether complementary policies (including same-day treatment initiation, task-shifting, reduced ART visits, and reduced ART pickups) implemented around the same time may have increased ART linkage.

**Methods:**

HIV testing and treatment data were collected by PEPFAR country programs in 13 African countries from 2015 to 2018. These countries were chosen based on the completeness of policy data and availability of program data during the study period. Program data were used to calculate proxy linkage rates. These rates were compared relative to the Treat All adoption period and the adoption of complementary policies.

**Results:**

The 13 countries experienced an average increase in ART linkage of 29.3% over the entire study period. In examining individual countries, all but two showed increases in linkage to treatment immediately after Treat All adoption. Across all countries, those that had adopted four or more complementary policies showed an average increased linkage of 39.8% compared to 13.9% in countries with fewer than four complementary policies.

**Conclusions:**

Eleven of 13 country programs examined in this study demonstrated an increase in ART linkage after Treat-All policy adoption. Increases in linkage were associated with complementary policies. When exploring new public health policies, policymakers may consider which complementary policies might also help achieve the desired outcome of the public health policy.

## Background

Since the first identified human cases in 1981, HIV has resulted in approximately 33 million AIDS-related deaths worldwide [1]. The advent of antiretroviral therapy (ART) in 1996 was a lifeline for people living with HIV (PLHIV). However, global economic inequity kept this medical advancement from reaching most persons who needed it. Over 99% of PLHIV in low- and middle-income countries lacked access to HIV treatment in 2000 [2].

In the early 2000s, global access to HIV testing and treatment began to be addressed with establishing the Global Fund to Fight AIDS, TB, and Malaria in 2002 and the United States President’s Emergency Plan for AIDS Relief (PEPFAR) in 2003. Since then, the Global Fund and PEPFAR have contributed over one hundred billion U.S. dollars to prevent and treat HIV worldwide, saving millions of lives [3]. As of 2020, PEPFAR supports HIV treatment for 18.96 million of the 37.7 million PLHIV globally [1, 4]. Still, a treatment gap persists as some 9.5 million PLHIV are not on treatment [5].

In 2015, the World Health Organization (WHO) recommended immediate initiation of HIV antiretroviral therapy (ART) for all PLHIV [6] regardless of immune status or risk group. The guidelines provided recommendations to start PLHIV on ART earlier, implement differentiated approaches, and improve the quality and efficiency of services to achieve the UNAIDS 90-90-90 treatment target by the end of 2020 and the 95-95-95 target by 2030. This recommendation came on the heels of clinical trials finding significant treatment and prevention benefits of starting ART upon diagnosis [7, 8] and has been referred to by various terms such as Test and Treat, Universal Test and Treat, Treat-All, and Test and Start, which acknowledges lifelong treatment utilized to maintain a low viral load and prevent onward transmission. We will use the term Treat-All, throughout this manuscript.

By July 2019, 93% of low- and middle-income countries (LMICs) adopted a Treat-All policy [9]. There are several studies that have looked at effects of Treat All implementation on healthcare practices and CD4 and viral load testing, with mixed findings [10, 11].

We sought to describe the rate of ART linkage before and after the adoption of a Treat-All policy in 13 PEPFAR-supported countries in Africa: Botswana, Cameroon, Ethiopia, Kenya, Lesotho, Malawi, Mozambique, Namibia, Nigeria, Rwanda, Tanzania, Zambia, and Zimbabwe. We examined 1) linkage rates to ART initiation over time before and after the adoption of Treat-All in each country; 2) how Treat-All implementation differed across these countries; and 3) whether complementary policies (such as same-day treatment initiation, task-shifting from doctors to nurses, task-shifting from nurses to community health workers, reduced ART visits, and reduced ART pickups) implemented around the same time may have increased ART linkage.

## Methods

### Data sources

PEPFAR Monitoring, Evaluation, and Reporting (MER) indicators were used to assess ART linkage initiation over time before and after adopting Treat-All. MER is a strategic information framework that monitors program outputs, outcomes, and programmatic impact [12]. The indicators are a comprehensive list of indicators reported by PEPFAR-funded implementing partners to PEPFAR and CDC country offices on a quarterly, semi-annual, and annual basis during the United States Government fiscal year (F.Y.) that begins on October 1st of the prior calendar year. Two quarterly MER indicators were used: the number of people who tested positive for HIV in a given quarter and the number of adults and children newly enrolled on ART in a given quarter. These indicators are aggregate counts at the facility level and do not necessarily include the same people; direct calculation of ‘true’ linkage cannot be obtained using these variables. Instead, a proxy linkage rate is calculated. In the results, the proxy linkage is referred to as linkage and the referenced year refers to the fiscal year.

The linkage to care proxy was calculated using the MER data and the below formula:

$$Proxy\;Linkage=\frac{Number\;of\;people\;newly\;on\;ART\;in\;Quarter\;X}{Number\;of\;people\;tested\;positive\;for\;HIV\;in\;Quarter\;X}$$

Policy data were collected from two PEPFAR sources: PEPFAR Policy Tracking Tables (PTTs) (from F.Y. 16) and the PEPFAR Sustainability Index and Dashboard (SID) (from F.Y.s 15, 17, and 19) [13, 14]. The PTTs tracked progress on adoption and implementation of HIV-related policies, including Treat-All, and were used to monitor policy reforms over five distinct stages of the policy cycle: (1) Identify Baseline Policy Issue(s)/Problem(s); (2) Develop Policy Intervention and Document; (3) Official Government Endorsement of Policy; (4) Implement Policy; and (5) Evaluation of Policy Impact on Health. The PTT for FY16 was used for this analysis as that is the fiscal year most countries adopted Treat-All policies [13].

The SID measures the sustainability of national HIV/AIDS programming across 15 elements, is completed by PEPFAR staff and in-country stakeholders, and is submitted every other fiscal year. SIDs contain information about complementary policies adopted (or not adopted) around the same time as Treat-All policies. The complementary policies are same-day treatment initiation, task-shifting from doctors to nurses, task-shifting from nurses to community health workers, reduced ART visits, and reduced ART prescription pickups (where enough ART medication is provided for multiple months, rather than the standard 30 days; also known as Multi-Month Dispensing). In addition, the SIDs collect the percentages of host government funding contributions to ARVs and HIV test kits.

### Country selection

Although PEPFAR supports fifty country and regional HIV/AIDS programs throughout the world, countries were only included in the analysis if they met all the following criteria:

1. Availability of an FY16 PTT
2. MER data available 1-year pre- and post-policy adoption
3. More than 50% of data completion from FY16 PTT

**Fig. 1:**
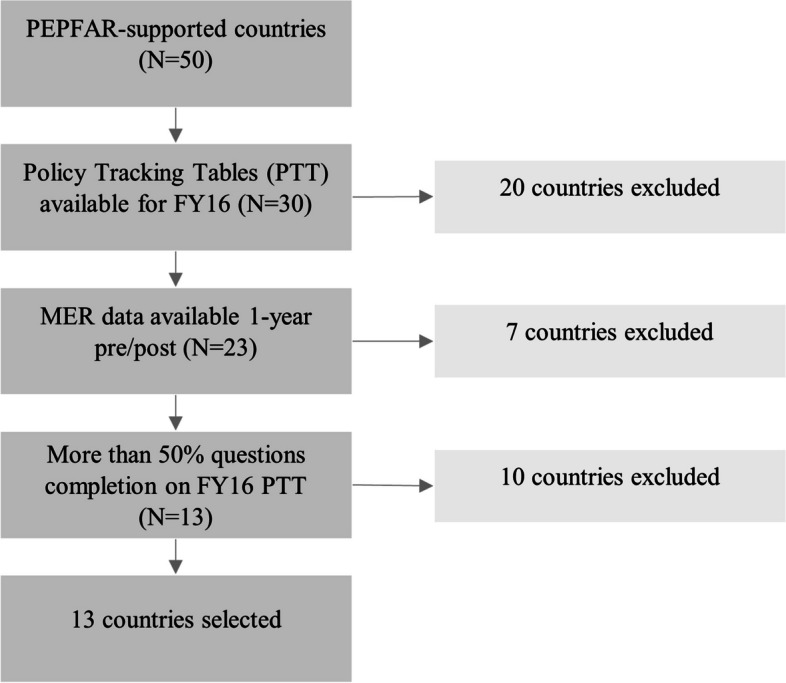
PEPFAR country inclusion and exclusion criteria and resulting decisions

Figure 1 shows inclusion criteria and the resulting decisions about countries for analysis. Thirteen countries were included for analysis.

### Analysis

Treat-All policies were adopted from FY16 Q2 to FY17 Q2, and ART proxy linkage rates were examined one year preceding and following the Treat-All policy adoption of the country, resulting in different one-year periods for each country. Proxy linkage was calculated (see above) for each country and overall, by quarter. We also calculated proxy linkage within categories of the number of complementary policies implemented. All analyses were completed using SAS 9.4 [15] and Excel 2016.

Qualitative implementation metrics were compiled into a comprehensive Excel database and examined for themes. The themes included adopting strategies such as conducting formal analyses, establishing technical working groups, and implementing strategies like dissemination strategies for subnational policymakers and healthcare workers and resource costing and implementation.

From the SIDs collected in 2017, five complementary policies and two funding metrics were examined. Countries were assigned a binary score for each complementary policy (1 = Yes, policy was in place; 0 = No, policy was not in place). Countries were also assigned a binary score for funding metrics based on the amount the host government funded of either ARVs (funding metric 1) or HIV testing kits (funding metric 2) (1 = 50% or more funded by the host government; 0 = Less than 50% funded by host government). These scores were summed to give a total score on complementary policies. The total scores were then dichotomized using a cut-off of 4. Countries with a total score greater than 4 were considered to have high levels of complementary policies, and those with less than 4, had low levels of complementary policies. Individual and total scores were used in the analysis.

## Results

### Overview

The countries that met the criteria for inclusion were Botswana, Cameroon, Ethiopia, Kenya, Lesotho, Malawi, Mozambique, Namibia, Nigeria, Rwanda, Tanzania, Zambia, and Zimbabwe (*n* = 13). One country program, Tanzania, had an incomplete MER dataset for the four years, and the associated fiscal year (FY15) was excluded from the analysis. All countries adopted Treat-All policies between the second quarter of FY16 to the second quarter of FY17. Ten countries adopted Treat-All policies in FY16. Ethiopia, Tanzania, and Namibia adopted Treat-All in FY17.

### Adoption and implementation

All country programs reported the adoption of Treat-All policies by host country governments. In addition, all country programs reported that stakeholder meetings between the national government, PEPFAR, and non-governmental organizations were held prior to adoption. Eight (62%) country programs reported that a formal technical working group was created prior to policy adoption, and 6 (46%) country programs indicated that a formal analysis was undertaken prior to policy adoption. Five (38%) country programs reported that dissemination occurred through regional health offices to lower health system levels after the policy was adopted. Four (31%) country programs reported training for local health workers on policy implementation. All country programs noted that PEPFAR provided technical assistance during the adoption of Treat-All policies, and 9 (69%) country programs noted that PEPFAR was responsible for funding some or all Treat-All implementation at lower levels of the health system.

### Proxy linkage rates

**Fig. 2:**
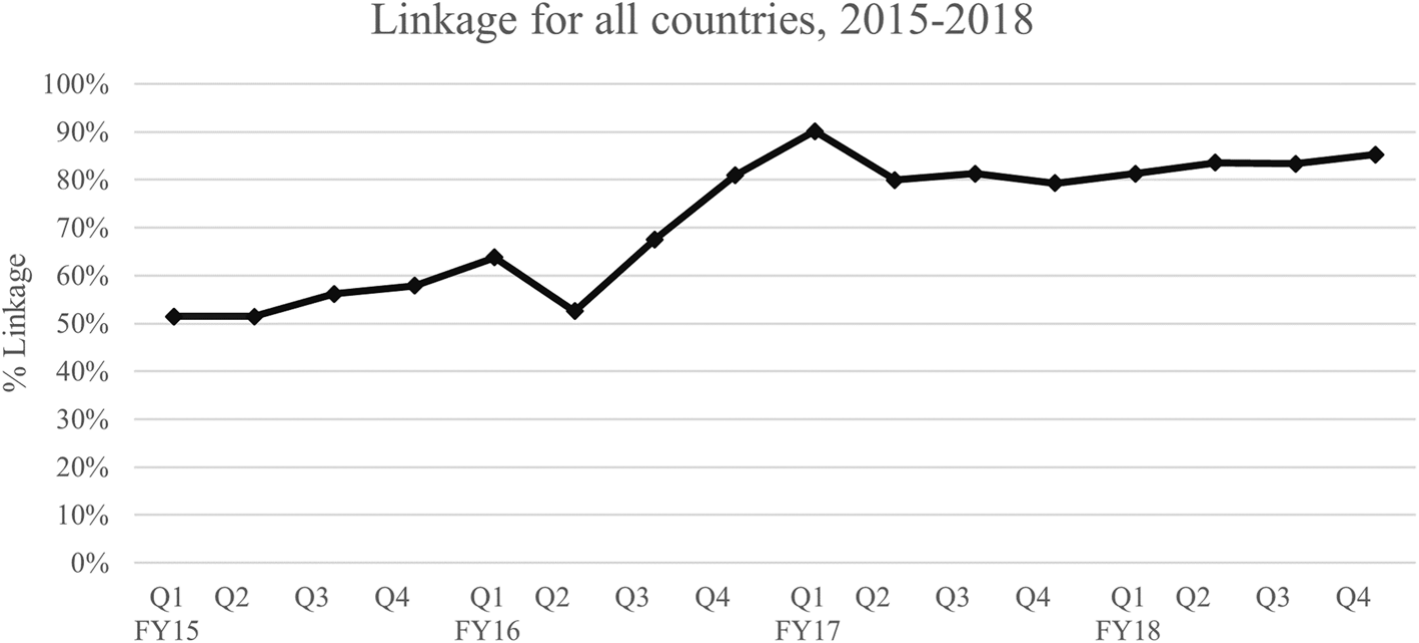
The 13 study countries reported linkage rate increases of 29.3% from 2015 to 2018

As seen in Fig. 2, between FY16 and FY18, the total linkage for all countries increased by 17.7%, with an average increase of 29.3%. In two countries, Ethiopia and Rwanda, the linkage rate decreased between FY15 and FY18. Six (46%) countries had linkage rate increases above 29.3%. The highest linkage rate increases were in Botswana and Malawi, which reported linkage rates above 100% in FY17 and FY18 (Table 1).

**Table 1:** TX_NEW, HTS_POS, and linkage rates for countries (2015–2018)

Country	FY15			FY16			FY17			FY18		
	%	n	N	%	n	N	%	n	N	%	n	N
Botswana	0.9	70	7820	64.4	10,802	16,783	124.3	19,252	15,490	103.9	21,020	20,232
Cameroon	81.9	15,966	19,498	77.2	39,372	50,982	82.9	43,394	52,336	93.4	37,213	39,849
Ethiopia	82.6	46,032	55,734	83.5	35,145	42,110	79.6	47,974	60,247	70.9	42,846	60,428
Kenya	68.2	165,505	242,836	73.3	176,507	240,885	78.8	149,963	190,247	78.9	145,122	183,866
Lesotho	63	27,152	43,067	81.2	30,830	37,974	90.9	33,610	36,965	95.1	30,297	31,873
Malawi	62.2	97,671	157,070	79.9	112,871	141,345	194.3	119,549	61,543	139.9	118,811	84,901
Mozambique	42.3	204,646	483,831	55	227,576	414,141	80.9	317,171	391,879	82.7	335,470	405,850
Namibia	21.2	2409	11,376	90.8	13,986	15,403	94.7	19,301	20,389	82.6	17,924	21,695
Nigeria	45.4	126,188	278,218	60.6	146,352	241,465	76.6	162,614	212,201	83.7	134,941	161,270
Rwanda	92.9	11,762	12,663	99.1	12,829	12,943	87.2	7693	8822	90.5	7681	8485
Tanzania	N/A	N/A	N/A	70.1	189,270	269,993	78.1	249,124	318,786	75.1	245,847	327,542
Zambia	56.1	130,032	231,891	60.2	120,473	200,152	67.1	165,396	246,559	78.9	210,132	266,227
Zimbabwe	62.1	62,428	100,597	59.4	90,330	151,980	79.1	128,974	162,949	81.6	119,583	146,537
All	54.1	889,861	1,644,601	65.7	1,206,343	1,836,156	82.3	1,464,015	1,778,413	83.4	1,466,887	1,758,755

Ten countries adopted Treat-All policies in FY16. Ethiopia, Tanzania, and Namibia adopted Treat-All in FY17

On average, country programs reported an increase of 24.3% between the preceding and subsequent years of Treat-All adoption. The highest linkage rate increases were in Botswana (76.6%), Malawi (77.1%), and Rwanda (41.5%). Kenya and Lesotho reported increased linkage rates between 20 and 30%. Mozambique, Namibia, Zimbabwe, and Nigeria all reported increases in linkage rates between 10 and 20%. Zambia’s linkage rate increased by 2.4%. Cameroon (-7.3%) and Ethiopia (-3.9%) reported a decrease in linkage rate between the two years.

### Complementary policies

Table 2 presents complementary policies implemented by country according to the 2017 SID. On average, countries reported between two and three complementary policies with the most common policies being clinician task-shifting and reduced ART pickups. The funding policies were the least commonly reported. Lesotho reported the highest number of adopted complementary policies (six policies of seven possible) and had slightly above average linkage across the four-year period (32.1% compared to 29.3%). Namibia and Zimbabwe adopted five policies each and linkage rose 61.4% and 19.5% in those countries, respectively. Three countries (Kenya, Tanzania, and Zambia) did not have any reported complementary policies and their linkages increased by 10.7%, 5%, and 22.8%, respectively.

**Table 2:** Complementary policies enacted by country

Country	Task shifting Clinicians	Task shifting CHWs	ART reduced visits	ART reduced pickups	ART same day initiation	Percent of ARVs funded by host government > = 50%	Percent of test kits funded by host government > = 90%	Total score
Botswana	No	No	Yes	No	No	Yes	Yes	3
Cameroon	Yes	Yes	No	No	No	No	No	2
Ethiopia	Yes	No	No	Yes	No	No	No	2
Kenya	No	No	No	No	No	No	No	0
Lesotho	Yes	Yes	Yes	Yes	Yes	Yes	No	6
Malawi	Yes	No	Yes	Yes	Yes	No	No	4
Mozambique	Yes	No	No	Yes	Yes	No	No	3
Namibia	Yes	Yes	No	Yes	No	Yes	Yes	5
Nigeria	No	No	No	Yes	Yes	No	No	2
Rwanda	Yes	No	No	Yes	No	No	No	2
Tanzania	No	No	No	No	No	No	No	0
Zambia	No	No	No	No	No	No	No	0
Zimbabwe	Yes	Yes	Yes	Yes	Yes	No	No	5

**Fig. 3:**
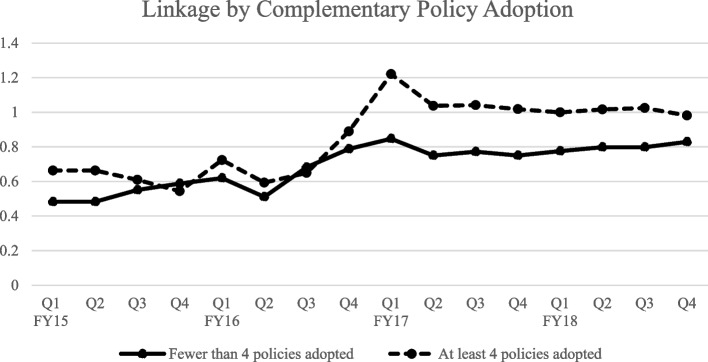
Linkage rates by number of complementary policies adopted

Four (31%) countries met the criteria of 4 or more complementary policies or financing mechanisms in place. In countries that met the criteria, a 39.8% increase was observed (Fig. 3). Countries that did not meet the criteria had a 13.9% increase observed.

In all cases, countries with four or more complementary policies had increased linkage rates compared to the countries fewer than four policies. However, the countries with fewer complementary policies had higher linkage rates at the beginning of the study period. Those rates changed more slowly than the countries with four or more complementary policies between 2015 and 2018.

## Discussion

In our analysis of 13 PEPFAR-supported countries in Africa, the overall linkage from HIV diagnosis to ART initiation increased after the Treat-All policy adoption (24.3% over two years and 29.3% over four years). Consistent trends were not seen among all countries. Eleven countries experienced increases in linkage while two experienced decreases in linkage over the entire four-year study period. Cameroon experienced a decrease in linkage in the year after Treat-All policy adoption but an overall increase in linkage over the four years. The differences in country data may reflect differences in country programming and reporting. For example, Lesotho adopted other practices concurrent to Treat All such as men-friendly and adolescent-friendly clinics, community ART teams working with mobile testing clinics, and linking ART services with maternal and TB services. In addition, the uptake of mobile technologies used for tracking and confirming linkage (particularly critical when testing and treatment are not done at the same location) may have improved linkage. Cameroon also worked with providers to provide improved pre-and post-test counselling, complete ART readiness assessments for patients, and to physically assist patients throughout the facility to ensure same-day linkage to care. In other circumstances, such as Namibia, patients often wanted time (from weeks to months) to process their HIV diagnoses and begin ART in subsequent quarters. In addition, patients would transfer to different facilities to start ART. Both processing diagnoses and switching facilities led to decreases in measured linkage. Other complementary programmatic policies were adopted, such as switching to a DTG-based regimen in Botswana that may have influenced the uptake of ART treatment by newly positive patients. Further mapping exercises to understand implementation and timing of complementary policies and their relation to linkage would benefit current understanding of how numerous policies tackling a similar challenge can affect change.

Not all fluctuation within a country can be explained by Treat-All policy adoption. Other in-country factors such as implementation and program quality and the site or funding-level decisions may have played a role in linkage changes. There was a large push in many of these countries to increase awareness of testing and HIV status or of treatment availability once Treat All was adopted. These community awareness campaigns likely also played a large role in people seeking testing and treatment during this period. Also, social or individual factors play a role in the willingness of individuals to start treatment immediately after receiving an HIV diagnosis. Individuals may want to re-take an HIV test at a different facility or take time to understand their diagnosis before starting treatment [16, 17]. In addition, asymptomatic patients may not perceive the need to start on ART after diagnosis [16]. Countries in this study varied in HIV prevalence during the study period, ranging from five countries with less than 5% prevalence of HIV in 2017, two countries with 5–10% prevalence, three countries with 10–20% prevalence, and two countries with over 20% prevalence [18]. Prevalence may influence individual health-seeking behaviour.

Countries reported similar experiences in adopting Treat-All policies in that eight countries had working groups formed, and all received funding from PEPFAR for implementation. However, after adoption, the Treat-All policy implementation varied widely between countries. Only five countries reported subnational dissemination and four countries reported performing healthcare worker training on Treat-All. This may be due to incomplete PTT data, which suggests a lack of understanding or reporting of how the policy was implemented after adoption or utilizing the PTT. While many PTTs had complete data for the policy adoption phase, there appeared to be less knowledge about how effectively the policy was implemented after adoption. Other studies of Treat-All implementation have shown that some sites began implementing the policy as quickly as less than one month after adoption, perhaps showing quick dissemination through informal or non-governmental channels [9].

The differences in linkage between countries with four or more complementary policies and those with fewer than four were greater than the differences between periods before and after Treat-all policy adoption. Across all countries, those who had adopted four or more complementary policies experienced higher increases in linkage from FY15 to FY18 than those who had fewer than four complementary policies. However, while those countries with fewer complementary policies started with higher linkage rates in FY15 than those with four or more complementary policies, by FY18, the linkage rates of countries with complementary policies had all surpassed those without these policies. Also, certain policies seemed to align with larger increases than others. These policies included same-day ART initiation, clinician task-shifting, and reduced ART pickups.

This study has several limitations. The largest limitation was the proxy indicator for linkage rather than the direct calculation of linkage. Once Treat All was adopted, some countries may have implemented specific programs to bring people onto treatment who had tested positive in a previous data collection period (quarter), creating a linkage rate over 100%. This may help explain country programs like Malawi and Botswana, where the linkage rate was over 100% over half of the data collection period. Secondly, the denominator in the linkage calculation is of HIV-positive tests, not HIV-positive persons. Therefore, there may be double-counting of HIV-positive persons in the denominator, artificially deflating the linkage rate. Health-seeking behaviours differ by country and the practice of taking multiple tests before starting treatment could differ by country as well. Country programs report that double-counting decreased as unique patient identifiers became more common during the study period, meaning that data in earlier years were more affected. As noted earlier, the differences in country programming and reporting and changes in reporting standards during the study period can lead to non-comparable country-specific results. Additional studies that focus on individual countries that may have access to linkage registrars or individual-level data would be helpful in understanding the linkage behaviour and its ties to policy change. However, these studies would likely need to be limited to individual countries as non-PEFPAR tools and practices differ immensely between countries. Also, implementation measures could have been completed and not recorded in the PTTs— data collection and reporting abilities varied by country, particularly in FY15. Lastly, the SID data collected in 2017 may not be representative of the entire time period.

Finally, because the included countries were not selected randomly and may not be representative of other PEPFAR countries, and because country programming and reporting differ significantly from country to country, we limit generalizability to the included countries.

## Conclusions

In summary, the countries examined in this study demonstrated an average increase in ART linkage after Treat-All policy adoption. These patterns appear to mirror trends previously explored in the literature. In addition, complementary policies were aligned with increases in ART linkage. When exploring new public health policies, policymakers may consider which complementary policies, if any, might also help achieve the desired outcome of the public health policy.

## Data Availability

Datasets generated from raw data and analysed for the current study are available upon request to the corresponding author. *The SID data used for the current study are publicly available at Country and Regional Operational Plans - United States Department of State *(https://www.state.gov/country-operational-plans/)*.* CDC cleared this publication and its use of data.

## Abbreviations

ART: Antiretroviral Therapy
FY: United States Government Fiscal Year
LMIC: Low- and middle-income country
MER: Monitoring, Evaluation, and Reporting
PEPFAR: U.S. President’s Emergency Plan for AIDS Relief
PLHIV: People Living with HIV
PTT: Policy Tracking Tables
SID: Sustainability Index and Dashboard
WHO: World Health Organization
